# Neural basis underlying the sense of coherence in medical professionals revealed by the fractional amplitude of low-frequency fluctuations

**DOI:** 10.1371/journal.pone.0288042

**Published:** 2023-06-30

**Authors:** Kota Kanda, Shisei Tei, Hidehiko Takahashi, Junya Fujino

**Affiliations:** 1 Department of Psychiatry and Behavioral Sciences, Graduate School of Medical and Dental Sciences, Tokyo Medical and Dental University, Bunkyo-ku, Tokyo, Japan; 2 Department of Psychiatry, Graduate School of Medicine, Kyoto University, Sakyo-ku, Kyoto, Japan; 3 Medical Institute of Developmental Disabilities Research, Showa University, Kita-karasuyama, Setagaya-ku, Tokyo, Japan; 4 Institute of Applied Brain Sciences, Waseda University, Tokorozawa, Saitama, Japan; 5 School of Human and Social Sciences, Tokyo International University, Kawagoe, Saitama, Japan; 6 Center for Brain Integration Research, Tokyo Medical and Dental University, Bunkyo-ku, Tokyo, Japan; Harvard Medical School, UNITED STATES

## Abstract

Although mitigating burnout has long been a pressing issue in healthcare, recent global disasters, including the COVID-19 pandemic and wars, have exacerbated this problem. Medical professionals are frequently exposed to diverse job-induced distress; furthermore, the importance of people’s sense of coherence (SOC) over work has been addressed to better deal with burnout. However, the neural mechanisms underlying SOC in medical professionals are not sufficiently investigated. In this study, the intrinsic fractional amplitude of low-frequency fluctuations (fALFF) were measured as an indicator of regional brain spontaneous activity using resting-state functional magnetic resonance imaging in registered nurses. The associations between participants’ SOC levels and the fALFF values within brain regions were subsequently explored. The SOC scale scores were positively correlated with fALFF values in the right superior frontal gyrus (SFG) and the left inferior parietal lobule. Furthermore, the SOC levels of the participants mediated the link between their fALFF values in the right SFG and the depersonalization dimension of burnout. The results deepened the understanding of the counter role of SOC on burnout in medical professionals and may provide practical insights for developing efficient interventions.

## Introduction

Burnout has become a critical issue in the healthcare system [1–4]. A large proportion of medical professionals show burnout symptoms that may lead to substance abuse, medical errors, and even suicide [5]. They are constantly exposed to various stressors, including patients’ suffering, steep hierarchies, as well as team conflicts [5, 6]. Since the COVID-19 pandemic, medical professionals have experienced higher levels of stress [7–9].

Meanwhile, recent studies emphasized the crucial role of a sense of coherence (SOC) over work in dealing with burnout [3, 10, 11]. An SOC refers to the ability to consider stressful situations as manageable, understandable, and meaningful, which may be nurtured by proper training and learning experiences [5, 12]. The SOC that one can exert over these stressors may have a substantial influence, not only on burnout but also on work performance, especially for less-experienced medical professionals [3, 13–15]. In this line, we also reported that decreased SOC levels were associated with increased depersonalization symptoms of burnout as well as enhanced feelings of empathic distress in registered nurses [12]. For a better understanding of this issue, studying the neural substrates of SOC in medical professionals should be informative.

Resting-state functional magnetic resonance imaging (RS-fMRI) can be a promising tool in investigating neural substrates of SOC. It is because peoples’ intrinsic brain activity may reflect their levels of learning/training and mental maturation, through self-referential mental activity [16–18]. RS-fMRI enables the assessment of stable neural characteristics by measuring spontaneous brain activity while avoiding potential confounders highlighted by task-induced fMRI [19, 20]. However, neural substrates for SOC in medical professionals are still unexplored in a resting state.

In this study, the fractional amplitude of low-frequency fluctuations (fALFF) was used to assess the intensity of spontaneous brain activity by examining the amplitude of hemodynamic oscillations [21, 22]. The fALFF approach has not only characterized psychological trait-level features but also prompted subsequent perceptual sensitivity and cognitive performance [23–25]. In particular, this approach has been utilized to investigate the neural mechanisms underlying the efforts undertaken to achieve long-term goals among people experiencing chronic stress in various professional fields [26, 27]. Furthermore, several previous studies evaluated the neurobiological bases of stress-induced clinical conditions, including depression and anxiety, using the fALFF approach [28–30]. Therefore, fALFF approach is a promising approach to investigate the neural substrates of SOC in medical professionals.

Previous studies have repeatedly shown that the frontoparietal regions are activated in various types of cognitively demanding tasks among medical professionals [31, 32]. Therefore, we predicted that the fALFF values in these brain areas would be associated with the SOC levels in registered nurses.

## Materials and methods

### Participants

Forty-one registered nurses were enrolled in this study. The sample size was determined based on previous fMRI studies on burnout [33, 34]. Four participants were excluded from the analysis because of excessive head motion during MRI scanning (see S1 File for details). Thus, the data of the remaining 37 participants were used in the analyses (Table 1). No participants met the criteria for any psychiatric disorder per the Structured Clinical Interview for DSM-IV Axis I Disorders (SCID I), and none had a history of head trauma, neurological illness, serious medical or surgical illness, or substance abuse. The recruitment of the participants and the original data analysis of this study were performed from 2012 to 2017. This study was approved by the Committee on Medical Ethics of Kyoto University and was conducted per the Code of Ethics of the World Medical Association. All participants gave their written informed consent to participate in the study. Further details are described in S1 File.

**Table 1:** Demographic characteristics of the participants.

	Total (n = 37)
Age (years): mean ± SD [min–max]	27.0 ± 3.9 [22–39]
Sex: male/female	11/26
Handedness: right/left	35/2
IQ: mean ± SD [min–max]	97.6 ± 8.2 [81–110]
Years of profession ^a^: mean ± SD [min–max]	5.1 ± 4.0 [1.0–17.5]

^a^ Data not available for one participant

### Psychological measures

The SOC was estimated using the SOC scale [10, 12]. The SOC scale estimates the degree of participants’ belief that the resources are available to one to meet the demands posed by the stressor, the degree of tendency in challenges, worthy of investment and engagement, and the degree of impression of an environment as structured, predictable, and explicable [10]. A higher SOC scale score represents a greater sense of coherence.

The severity of the depersonalization dimension of burnout was assessed using the Japanese version of the Maslach Burnout Inventory [MBI, 35, 36], which measured participants’ intensity of emotional detachment toward the recipients of one’s care and defensive coping [35, 37], with higher scores denoting more depersonalization. In our previous study, we reported that the “emotional exhaustion” subscales were not correlated with SOC levels [12]. In addition, “personal accomplishment” subscales were reported to reflect the employees’ personalities rather than the burnout dimension [38–40]. Therefore, the emotional exhaustion and personal accomplishment subscales of the MBI were not used in the main analyses of this study.

### MRI data acquisition, pre-processing and fALFF calculation

All participants underwent MRI scanning on a 3 T whole-body scanner coupled with an 8-channel phased-array head coil (Trio, Siemens, Erlangen, Germany). Image processing and fALFF calculation were performed using SPM (Wellcome Trust Center for Neuroimaging, London, UK) and DPARSFA toolbox (Data Processing Assistant for Resting-state fMRI Advanced Edition, http://rfmri.org/DPARSF) in MATLAB (MathWorks, Natick, MA, USA). Please see S1 File for details.

### Data analyses

To explore the brain regions where the fALFF values were associated with the SOC scale scores, we performed multiple regression analyses throughout the whole brain using a general linear model framework in SPM [41]. Age and sex were entered into the model as covariates of no interest. Based on the previous studies [e.g., 23, 42], we reported clusters that survived the family-wise error (FWE) correction for multiple comparisons with a cluster-level *p* < 0.05 (at voxel-level, uncorrected *p* < 0.005). The VOI function in SPM was used to extract the parameter estimates from the significant clusters. We then performed correlation analyses between the fALFF values and the depersonalization scores. If significant correlations were observed, we conducted mediation analyses to determine whether SOC mediates the relationship using the INDIRECT macro for SPSS [43]. We tested the significance of the mediation effect via a bootstrapping strategy within this macro. The mediated effect is considered statistically significant, if a confidence interval does not contain zero. In the current study, the threshold for statistical significance was set at *p* < 0.05 (two-tailed).

## Results

The participants’ SOC scale scores were 91–172 (mean ± SD = 126.2 ± 18.4). The depersonalization dimension of burnout scores were 6–20 (mean ± SD = 11.8 ± 3.5). The outcomes reflected considerable individual differences in these scores in our sample. The additional findings of emotional exhaustion and personal accomplishment subscales are detailed in S2 File, S1 Fig and S1 Table.

**Fig 1:**
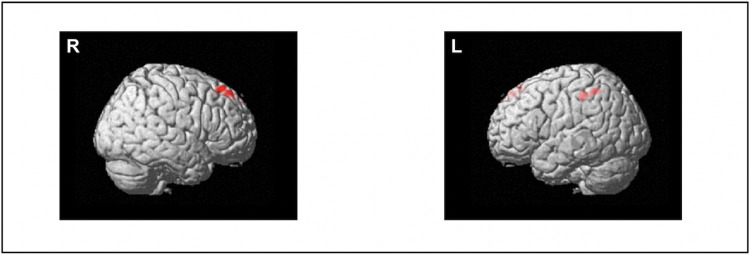
Brain regions showing spontaneous brain activity levels associated with SOC. A statistical threshold was set at cluster-level FWE-corrected *p* < 0.05. The SOC scale scores were positively correlated with fALFF values in the right superior frontal gyrus and the left inferior parietal lobule. Abbreviations: fALFF = fractional amplitude of low-frequency fluctuations, FWE = family-wise error, SOC = sense of coherence.

Fig 1 and S2 Table present the brain regions where fALFF values were correlated with SOC scale scores. The SOC scale scores were positively correlated with fALFF values in the right superior frontal gyrus (SFG) and the left inferior parietal lobule (IPL). In other words, participants with higher fALFF values in these areas showed higher SOC levels. We did not observe any significant areas where the fALFF values were negatively correlated with SOC scale scores.

**Fig 2:**
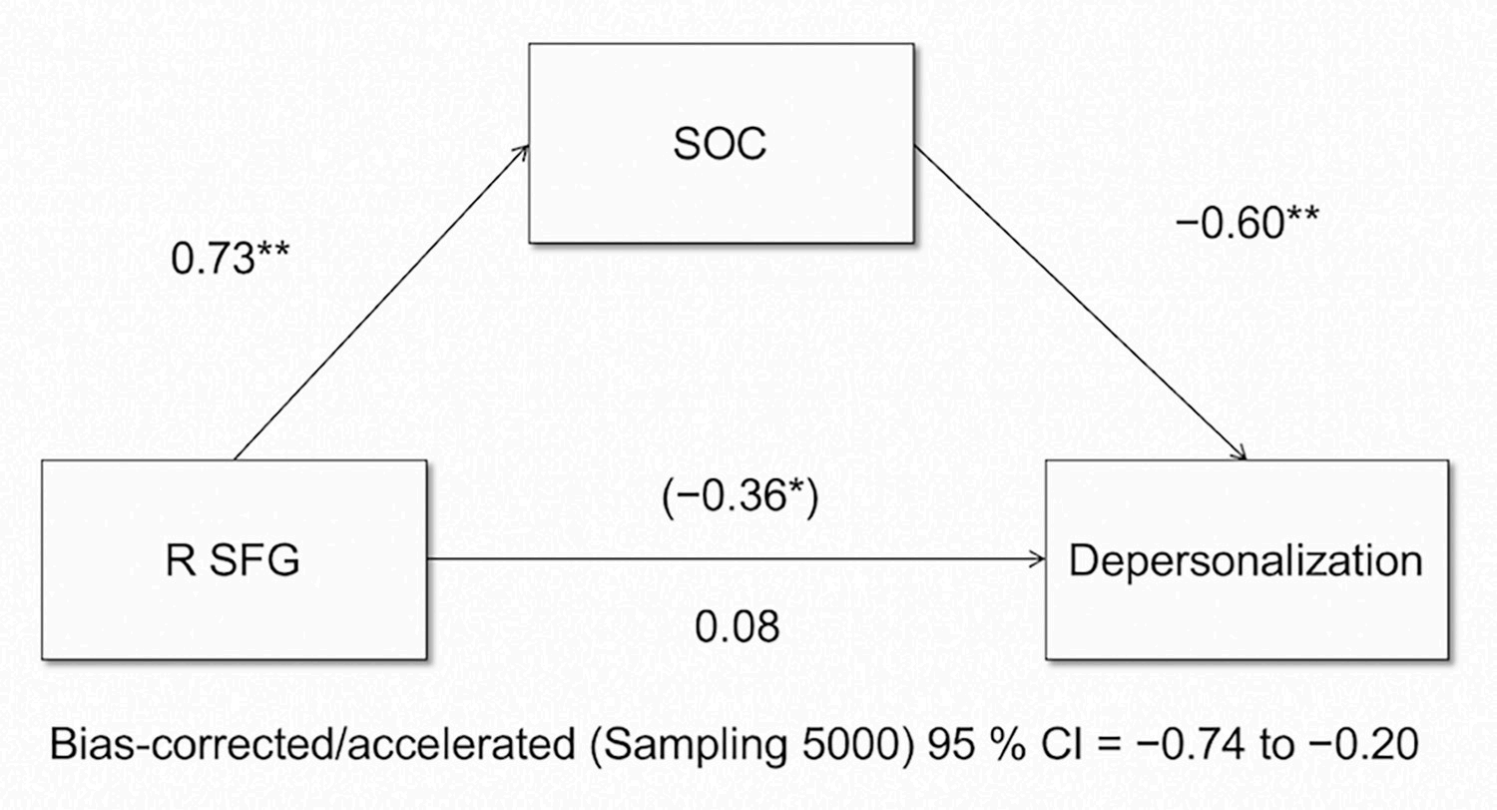
Mediation analysis. Mediation analysis found that the SOC mediated the relationship between the fALFF values in the R SFG and the depersonalization. Standardized coefficients and significance indicated by asterisks are reported for each path. *p < 0.05, **p < 0.01. Abbreviations: fALFF = fractional amplitude of low-frequency fluctuations, R = right, SFG = superior frontal gyrus, SOC = sense of coherence.

The level of depersonalization was negatively correlated with fALFF values in the right SFG (*r* = −0.36, *p* = 0.03), while this correlation was not evident in the left IPL (*r* = −0.29, *p* = 0.08) (S2 Fig). Then, we conducted mediation analyses to examine whether the SOC mediated the relationship between the fALFF values in the right SFG and depersonalization. The analyses revealed that the SOC was a significant mediator of the relationship (Fig 2).

## Discussion

To the best of our knowledge, this is the first report to examine the individual spontaneous neural activity associated with the levels of SOC in medical professionals.

Our results revealed that the fALFF values in the right SFG were positively correlated with SOC; i.e., nurses with higher fALFF values in the right SFG more frequently perceived stressors as controllable. The SFG is involved in various cognitive functions, including inhibition, working memory, and self-monitoring [44, 45]. Especially, this area plays a key role in cognitive control and the reappraisal of negative stimuli [28, 46, 47]. Our results suggest that greater spontaneous SFG activity may reflect more readiness [48, 49] and capacity in stress management via cognitive control/reappraisal [50]. This could be because spontaneous brain activity may implicate the energy consumption for maintaining the brain’s system ready at resting [20], and prompt subsequent cognitive performance when necessary [48, 51]. Along this line, the SFG activity showed an increase during cognitively demanding tasks such as clinical reasoning among medical residents [31, 32]. Taken together with those of previous studies, our findings suggest that the SFG is crucial in determining the capacity to regard stressful situations as manageable, understandable, and meaningful.

We also found that the fALFF values in the right SFG were associated with the depersonalization symptoms of burnout. Previous functional and structural MRI studies have repeatedly found that the dorsolateral prefrontal cortex, including the SFG, is associated with burnout severity [52, 53]. In line with the findings of these previous studies, the results highlight the crucial role of the SFG in burnout. Further, the mediation analysis showed that the SOC was a significant mediator of this relationship. One potential interpretation could be that the SOC may weaken depersonalization by prompting the optimistic reappraisal and understanding of stressful situations with a flexible shifting of perspectives [54, 55]. It may encourage the integration of conflicting stress cues into a coherent sense of experience. In effect, workplace stressors can be rather acknowledged as fulfilling/meaningful than as distressing; however, without sufficient SOC, frequent exposure to these stressors can lead to maladaptive coping.

In addition, the fALFF values in the left IPL were positively correlated with the SOC scale scores. The IPL is known to be involved in language processing, mathematical operations, the perception of facial emotion, and the interpretation of sensory information [56]. These functions are crucial in handling complex medical situations demanding verbal and nonverbal communication [57]. To this end, continued cultivation of such processing may develop into the maturity of SOC via the neuroplasticity effects of learning and experience. The current findings comprise practical hints for a better understanding of SOC and the prevention of burnout among medical professionals.

In summary, our results indicated that the levels of SOC among medical professionals can be indeed reflected in their resting-state brain activity. Participants’ levels of subjective well-being may be captured by the continuum of burnout to work-engagement with SOC as depicted in their spontaneous brain activity. To elaborate, burnout is considered one end of a continuum in the interpersonal relationship people establish at work in a maladaptive manner [37], and it contrasts with the opposite end (work engagement). In the interim, we noticed that fALFF values in the right SFG were linked to enhanced SOC and lessened depersonalization symptoms of burnout. In this viewpoint, individuals’ spontaneous SFG activity could implicate peoples’ SOC intensity that may weaken depersonalization subjectivity, and it may concurrently prompt engagement to work via the feeling of control, which further consolidates coherent experience to envision one’s past, present, and future experiences [58]. Recently, cognitive load theory is garnering more recognition in medical education; this theory draws upon the characteristics of working memory and long-term memory and the relationship between them to determine how people learn [59–61]. Cognitive load theory is of particular relevance to medical education owing to the tasks and professional activities that can be learned to necessitate the simultaneous integration of multiple sets of knowledge, behaviors, and skills at a specific time and place [59–62]. Future neuroimaging studies of SOC, alongside a cognitive load theory flamework, should aid us in better understanding how learners in the medical professions struggle with mastering complex concepts and developing toward expertise.

Recently, we reported that the structural brain correlates with the burnout severity in medical professionals using a voxel-based morphometric technique [4]. The results showed that the volumes of gray matter in the prefrontal cortices, including ventromedial prefrontal cortex, insula, and thalamus play a key role in the individual differences of burnout severity among medical professionals [4]. Combined with the findings of our previous study, the current RS-fMRI results for SOC provide useful information in developing effective interventions for burnout.

However, our study has several limitations that should be taken into consideration when interpreting its results. First, this is a cross-sectional study that precludes any interpretation of potential causality. To further examine the causal relationship between the strength of SOC and fALFF values, longitudinal studies should be conducted. In this endeavor, an interventional study promoting SOC should shed more light on this issue. Second, spontaneous brain activity was evaluated via fALFF in the context of functional segregation. Although this approach has been applied as a reliable method of localizing brain regions in relation to personality/behavioral traits [23, 24], future studies should combine the functional connectivity approach with fALFF to gain further insights into the network context [63]. In addition, studies repeatedly reported that gray matter density and white matter integrity were associated with individual differences in various types of social cognitive abilities in healthy subjects and clinical populations [64–67]. Future studies utilizing multimodal MRI should provide deeper insights into the SOC mechanisms among medical professionals. Finally, our sample consisted of only registered nurses. Thus, our study needs to be carefully re-examined and replicated with more participants from different occupational groups that varied in clinical experience levels.

Despite these limitations, the current study deepens our understanding of the neural underpinnings of SOC among medical professionals. Further studies on this subject will help determine the kinds of intervention and training that effectively impact the subjective experience of coherence/control in medical education.

## Supporting information

S1 File Participants, MRI data acquisition and pre-processing, fALFF calculation, supplementary references. (DOCX)

S2 File Additional results of emotional exhaustion and personal accomplishment subscales of the MBI, supplementary references. (DOCX)

S1 Fig Results of correlation analyses between SOC scale scores and subscales of MBI. (PDF)

S2 Fig Results of correlation analyses between depersonalization scores and fALFF values in the right SFG/left IPL. (PDF)

S1 Table Scores of SOC and MBI. (DOCX)

S2 Table Brain regions showing spontaneous brain activity levels associated with SOC. (DOCX)
