# Supplementary material for: Neural basis underlying the sense of coherence in medical professionals revealed by the fractional amplitude of low-frequency fluctuations
Source: PLoS One. 2023 Jun 30;18(6):e0288042. doi: 10.1371/journal.pone.0288042 (PMC10313006; doi:10.1371/journal.pone.0288042)
Supplement: S1 File — (DOCX) [file pone.0288042.s001.docx]

**Supporting Information**

**Neural basis underlying the sense of coherence in medical professionals revealed by the fractional amplitude of low-frequency fluctuations**

**Supplementary Methods**

***Participants***

Forty-one registered nurses were enrolled in this study. The sample size was determined based on previous fMRI studies on burnout [1, 2]. Owing to excessive head motion during MRI scanning, four participants were excluded from the study (see MRI data acquisition and pre-processing sections for details). Therefore, the data of the remaining 37 participants were utilized in the analyses. Our present study population comprised 91.9 % of the sample (34 participants) recruited in our prior behavioral study [3]. Nevertheless, the results of our current neuroimaging study used methodologically distinct analyses of the previous dataset.

No participants met the criteria for any psychiatric disorder per the Structured Clinical Interview for DSM-IV Axis I Disorders (SCID I), and none had a history of head trauma, neurological illness, serious medical or surgical illness, or substance abuse. The predicted IQ was estimated by the Japanese Version of the National Adult Reading Test [4]. Participants were recruited via advertisements in the hospitals.

This study was approved by the Committee on Medical Ethics of Kyoto University and was conducted per the Code of Ethics of the World Medical Association. All participants gave their written informed consent to participate in the study. This study is a part of a larger project aimed at assessing individual differences between medical professionals, and participants completed a range of measures, not all of which are reported here.

***MRI data acquisition and pre-processing***

The RS-fMRI scanning schedule was arranged so that no participants were seriously sleep-deprived on the night before the scanning, to avoid the acute effects of sleep deprivation. During the scanning, participants were asked to keep their eyes open and passively stay focused on a fixation cross back-projected onto a screen. Functional images were captured using a T2*-weighted gradient-echo echo-planar imaging (EPI) sequence employing a 3T MRI scanner equipped with an eight-channel phased-array head coil (Trio, Siemens, Erlangen, Germany). The image acquisition parameters were as follows: repetition time (TR) = 2000 ms, echo time (TE) = 30 ms, field of view (FOV) = 256 × 192 mm, matrix = 64 × 48, and 30 interleaved axial slices of 4-mm thickness without gaps. The first two volumes were not saved to enable signal stabilization and the subsequent 180 volumes were obtained. Structural scans were also obtained using T1-weighted 3-dimensional magnetization-prepared rapid gradient echo (3D-MPRAGE) sequences (TR = 2000 ms, TE = 4.38 ms, inversion time = 990 ms, FOV = 225 × 240 mm, matrix = 240 × 256, resolution = 0.9375 × 0.9375 × 1.0 mm^3^, and 208 total axial sections without intersection gaps).

Image processing was performed using SPM8 (Wellcome Trust Center for Neuroimaging, London, UK) and the DPARSFA toolbox (Data Processing Assistant for Resting-state fMRI Advanced Edition, <http://rfmri.org/DPARSF>) in MATLAB (MathWorks, Natick, MA, USA).The skin–skull-stripping procedure for each subject was performed using the brain extraction tool from FSL (http://www.fmrib. ox.ac.uk/fsl/).

Images were manually oriented to the AC-PC line. Functional images were modified for differences in slice-acquisition timing and were then spatially realigned to correct head motion. T1-weighted images were co-registered to EPI images and segmented into gray matter, white matter, and cerebrospinal fluid. To reduce the effects of physiological processes and head motion, we regressed out the motion parameters, white matter signal, cerebrospinal fluid signal, and global signal. The global signal is considered to reflect a combination of physiological processes (such as cardiac and respiratory fluctuations); thus, it was treated as a covariate to regulate such factors. Although there is an ongoing debate about whether regressing out the global signal is advantageous or affects data detrimentally [5], we applied global signal regression because prior reports have also demonstrated that the global signal regression is effective in controlling movement-related artifacts in RS-fMRI data assessment [6, 7]. Functional images were spatially normalized to the Montreal Neurological Institute space (voxel size: 2 × 2 × 2 mm) and then smoothed using a Gaussian kernel with a full width at a half-maximum of 6 mm in the x, y, and z axes. Based on the previous studies [8], four participants who exhibited head motions of more than 1.5 mm or 1.5 rotations (max) during the scanning were excluded.

***fALFF calculation***

The fALFF calculation was performed using the DPARSFA toolbox. Based on previous studies [9, 10], the fALFF value of each voxel was computed for each participant. The sum of the amplitudes within a low-frequency range (0.01–0.1 Hz) was extracted, for the time series of each voxel. The fALFF was then calculated as the fractional sum of the amplitudes within the low-frequency range divided by the sum of amplitudes across the entire frequency range [9, 10]. Per prior studies [11, 12], the fALFF of each voxel was divided by the global mean fALFF value to standardize data across participants (the values were also obtained using the DPARSFA toolbox).

**Supplementary References**

[1] de Andrade AP, Amaro E, Jr., Farhat SC, Schvartsman C. Higher burnout scores in paediatric residents are associated with increased brain activity during attentional functional magnetic resonance imaging task. Acta Paediatr. 2016;105(6):705-713.

[2] Tei S, Becker C, Kawada R, Fujino J, Jankowski KF, Sugihara G et al. Can we predict burnout severity from empathy-related brain activity? Transl Psychiatry 2014;4:e393.

[3] Tei S, Becker C, Sugihara G, Kawada R, Fujino J, Sozu T et al. Sense of meaning in work and risk of burnout among medical professionals. Psychiatry Clin Neurosci. 2015;69:123-124.

[4] Matsuoka K, Uno M, Kasai K, Koyama K, Kim Y. Estimation of premorbid IQ in individuals with Alzheimer’s disease using Japanese ideographic script (Kanji) compound words: Japanese version of National Adult Reading Test. Psychiatry Clin Neurosci. 2006;60:332-339.

[5] Wong CW, Olafsson V, Tal O, Liu TT. Anti-correlated networks, global signal regression, and the effects of caffeine in resting-state functional MRI. NeuroImage. 2012;63:356-364.

[6] Klein C, Hiestand T, Ghadri JR, Templin C, Jancke L, Hanggi J. Takotsubo Syndrome-Predictable from brain imaging data. Sci Rep. 2017;7:5434.

[7] Power JD, Barnes KA, Snyder AZ, Schlaggar BL, Petersen SE. Spurious but systematic correlations in functional connectivity MRI networks arise from subject motion. NeuroImage. 2012;59;2142-2154.

[8] Son S, Miyata J, Mori Y, Isobe M, Urayama SI, Aso T et al. Lateralization of intrinsic frontoparietal network connectivity and symptoms in schizophrenia. Psychiatry Res Neuroimaging. 2017;260,23-28.

[9] Cox CL, Uddin LQ, Di Martino A, Castellanos FX, Milham MP, Kelly C. The balance between feeling and knowing: affective and cognitive empathy are reflected in the brain's intrinsic functional dynamics. Soc Cogn Affect Neurosci. 2012;7:727-737.

[10] Yu M, Xu M, Li X, Chen Z, Song Y, Liu J. The shared neural basis of music and language. Neuroscience. 2017;357:208-219.

[11] Lai CH, Wu YT. The patterns of fractional amplitude of low-frequency fluctuations in depression patients: the dissociation between temporal regions and fronto-parietal regions. J Affect Disord. 2015;175:441-445.

[12] Wei L, Duan X, Zheng C, Wang S, Gao Q, Zhang Z, et al. Specific frequency bands of amplitude low-frequency oscillation encodes personality. Hum Brain Mapp. 2014;35:331-339.
