# Supplementary material for: Neural basis underlying the sense of coherence in medical professionals revealed by the fractional amplitude of low-frequency fluctuations
Source: PLoS One. 2023 Jun 30;18(6):e0288042. doi: 10.1371/journal.pone.0288042 (PMC10313006; doi:10.1371/journal.pone.0288042)
Supplement: S2 File — (DOCX) [file pone.0288042.s002.docx]

**Supporting Information**

**Neural basis underlying the sense of coherence in medical professionals revealed by the fractional amplitude of low-frequency fluctuations**

**Supplementary Results**

***Additional results of emotional exhaustion and personal accomplish subscales of the Maslach Burnout Inventory (MBI)***

The emotional exhaustion subscales assessed feelings of being emotionally exerted and exhausted by one’s work with higher scores depicting more emotional exhaustion [1, 2]. The personal accomplishment subscales evaluated the subject’s sense of personal achievement at work with higher scores representing more personal achievement [1, 2].

The scores of emotional exhaustion and personal accomplishment subscales were 5–24 (mean ± SD = 16.8 ± 4.6) and 7–27 (mean ± SD = 15.5 ± 4.3), respectively (S1 Table). The levels of sense of coherence (SOC) were not significantly correlated with these subscales (emotional exhaustion: *r* = −0.28, *p* = 0.09, personal accomplishment: *r* = 0.20, *p* = 0.23) (S1 Fig).

**Supplementary References**

[1] Maslach C. Burnout: The Cost of Caring. Prentice Hall Trade: Englewood Cliffs, NJ, USA, 1982.

[2] Kubo M, Tao M. Burnout among nurses-the relationship between stresses and burnout. J Exp Soc Psychol. 1994;34:33-43.
