# Supplementary material for: Neural basis underlying the sense of coherence in medical professionals revealed by the fractional amplitude of low-frequency fluctuations
Source: PLoS One. 2023 Jun 30;18(6):e0288042. doi: 10.1371/journal.pone.0288042 (PMC10313006; doi:10.1371/journal.pone.0288042)
Supplement: S2 Fig — (PDF) [file pone.0288042.s004.pdf]

Supporting Information

Neural basis underlying the sense of coherence in medical professionals revealed by the fractional amplitude of low-frequency fluctuations

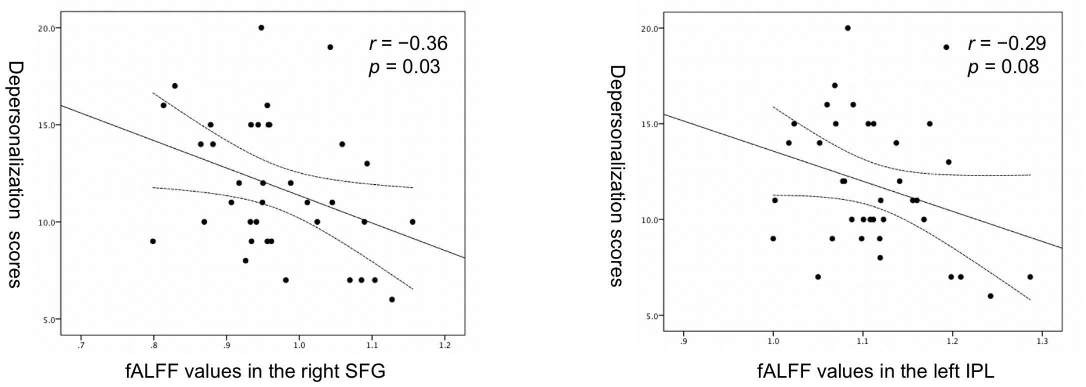

S2 Fig. Results of correlation analyses between depersonalization scores and fALFF values in the right SFG/left IPL. Dashed lines are 95% confidence interval boundaries. Abbreviation: fALFF = fractional amplitude of low-frequency fluctuations, IPL = inferior parietal lobule, SFG = superior frontal gyrus
