# Supplementary material for: Neural basis underlying the sense of coherence in medical professionals revealed by the fractional amplitude of low-frequency fluctuations
Source: PLoS One. 2023 Jun 30;18(6):e0288042. doi: 10.1371/journal.pone.0288042 (PMC10313006; doi:10.1371/journal.pone.0288042)
Supplement: S1 Table — (DOCX) [file pone.0288042.s005.docx]

**Supporting Information**

**Neural basis underlying the sense of coherence in medical professionals revealed by the fractional amplitude of low-frequency fluctuations**

**S1 Table. Scores of SOC and MBI.**

|  | Total (n = 37) |
| --- | --- |
| SOC: mean ± SD [min–max] | 126.2 ± 18.4 [91–172] |
| MBI |  |
| Depersonalization: mean ± SD [min–max] | 11.8 ± 3.5 [6–20] |
| Emotional exhaustion: mean ± SD [min–max] | 16.8 ± 4.6 [5–24] |
| Personal accomplishment: mean ± SD [min–max] | 15.5 ± 4.3 [7–27] |

Abbreviation: MBI = Maslach Burnout Inventory, SOC = sense of coherence
