# Supplementary material for: Neural basis underlying the sense of coherence in medical professionals revealed by the fractional amplitude of low-frequency fluctuations
Source: PLoS One. 2023 Jun 30;18(6):e0288042. doi: 10.1371/journal.pone.0288042 (PMC10313006; doi:10.1371/journal.pone.0288042)
Supplement: S2 Table — (DOCX) [file pone.0288042.s006.docx]

**Supporting Information**

**Neural basis underlying the sense of coherence in medical professionals revealed by the fractional amplitude of low-frequency fluctuations**

**S2 Table. Brain regions showing spontaneous brain activity levels associated with SOC.**

| Brain Region | Coordinates (mm) | | | *T* | Cluster |
| --- | --- | --- | --- | --- | --- |
|  | x | y | z |  | (voxels) |
| *Positive* |  |  |  |  |  |
| R superior frontal gyrus | 18 | 42 | 50 | 5.84 | 243 |
|  | 18 | 32 | 54 | 5.11 |  |
|  | 10 | 56 | 46 | 3.61 |  |
| L inferior parietal lobule | −40 | −34 | 46 | 4.90 | 217 |
|  | −36 | −52 | 52 | 3.72 |  |
|  | −40 | −44 | 50 | 3.69 |  |
|  |  |  |  |  |  |
| *Negative* |  |  |  |  |  |
| None |  |  |  |  |  |

*p* < 0.05, cluster-level FWE corrected (at voxel-level, uncorrected *p* < 0.005)

Three local maxima more than 8.0 mm apart are reported.

The coordinates are in the MNI space.

We interpreted the anatomical location of the clusters by consulting the Talairach Daemon database (http:// www.talairach.org), the Anatomic Automatic Labeling toolbox [1], and neuroanatomy atlas books [2, 3].

Abbreviations: FWE = family-wise error, L = left, MNI = Montreal Neurological Institute, R = right, SOC = sense of coherence.

**Supplementary References**

[1] Tzourio-Mazoyer N, Landeau B, Papathanassiou D, Crivello F, Etard O, Delcroix N et al. Automated anatomical labeling of activations in SPM using a macroscopic anatomical parcellation of the MNI MRI single-subject brain. NeuroImage. 2002;15:273-289.

[2] Duvernoy HM. The Human Brain. Surface, Three-dimensional Sectional Anatomy and MRI. Springer-Verlag, Wien, 1991.

[3] Talairach J, Tournoux P. Co-planar Stereotaxic Atlas of the Human Brain: 3-Dimensional Proportional System-An Approach to Cerebral Imaging. Thieme, New York, 1998.
